# Fast-track extubation in minimally invasive cardiac surgery: limits and lessons of a 4-year single-center analysis

**DOI:** 10.3389/fcvm.2025.1567533

**Published:** 2025-09-08

**Authors:** Sebastian Johannes Bauer, Moritz Benjamin Immohr, Friederike Irmgard Schoettler, Yukiharu Sugimura, Arash Mehdiani, Matthias Thielmann, Ajay Moza, Anna Fischbach, Michael Knapen, Evangelos Karasimos, Georg Eberhardt, Gereon Schaelte, Rolf Rossaint, Gernot Marx, Payam Akhyari

**Affiliations:** ^1^ Department of Thoracic- and Cardiovascular Surgery, West German Heart and Vascular Center, University Hospital Essen, Essen, Germany; ^2^ Department of Cardiac Surgery, University Hospital RWTH Aachen, Aachen, Germany; ^3^ Department of Anesthesiology, University Hospital RWTH Aachen, Aachen, Germany; ^4^ Department of Operative Intensive Care and Intermediate Care, University Hospital RWTH Aachen, Aachen, Germany

**Keywords:** minimally invasive cardiac surgery, extubation strategies, fast-track, valvular heart disease, enhanced recovery after surgery

## Abstract

**Background:**

Fast-track extubation is a key component of the interdisciplinary treatment concept Enhanced Recovery After Surgery (ERAS). In preparation for implementing ERAS as a comprehensive approach, we aimed to analyze the current state of fast-track extubation in the operating room, focusing on Minimally Invasive Cardiac Surgery (MICS). Specifically, we assessed the potential benefits of immediate on-table extubation compared to extubation within six hours after the completion of MICS.

**Methods:**

During a 4-year period from 2019–2023, a total of *n* = 146 patients underwent MICS at our institution. Surgical aspects were retrospectively analysed along with patients' risk profiles and relevant comorbidities. After 1:1 best neighbor propensity score matching, patients who were admitted to intensive care unit intubated but were extubated within six hours after surgery (fast-track, FT) were compared to those who were extubated in the operating room (extubation in tabula, EIT). The primary endpoint was fast-track failure (FTF), a composite of setbacks in the postoperative course: revision surgery, re-intubation, and readmission to ICU or intermediate care unit (IMC).

**Results:**

Patients had a median age of 61 years (IQR: 51.3–67.8) and were predominantly male (76.7%). The primary study endpoint occurred in 20.0% of all matched patients (FT: 26.7%, EIT: 13.3%; *p* = 0.289). FT patients had longer cardiopulmonary bypass times [FT 165.0 min (146.5–217.5); EIT 158.5 min (128.0–189.5); *p* = 0.047], but the duration of surgery was comparable. Additionally, the average length of hospital stay did not differ. A multivariate analysis was conducted and identified preoperative atrial fibrillation and intraoperative hypothermia as predictive risk factors for FTF.

**Conclusions:**

According to our retrospective single-center analysis, extubation in the operating room is feasible and safe even outside of a structured ERAS program. However, as itself it does not impact the further hospital stay, if there is no action thereafter, e.g., same day physiotherapy.

## Introduction

Minimally invasive cardiac surgery (MICS) is steadily replacing median sternotomy in cardiac surgery, especially for mitral valve surgery (1). In Germany, nearly 60% of single mitral valve surgeries use this approach (1), offering benefits like smaller incisions, reduced blood loss, and no risk of sternal instability (2). Akowuah et al. showed that although physical recovery at 12 weeks was similar, mini thoracotomy significantly shortened hospital stays, with twice as many patients discharged early (3).

Enhanced Recovery After Surgery (ERAS) also aims for early discharge and return to daily activities enhancing patient satisfaction by optimizing preoperative, intraoperative, and postoperative care to reduce postoperative morbidity and mortality. Certain measures should be highlighted: Preoperatively, adjusting glycated hemoglobin (HbA1c), albumin, and prehabilitation are recommended. Intraoperatively, body temperature should stay below 37.9°C during rewarming. Postoperatively, key practices include effective pain management with regional plane blocks, delirium screening, chest tube patency, and extubation within six hours to reduce intensive care unit (ICU) stay and hospital length of stay (4, 5). The ERAS Cardiac Society has issued recommendations on the timing of extubation in cardiac surgery (4–6). However, data on extubation in the operating room (OR) in patients undergoing MICS remain limited (7, 8). Furthermore, predictive preoperative risks for reversals in the postoperative course (fast-track failure, FTF)—such as revision surgery, re-intubation, and readmission to ICU or intermediate care unit (IMC)—have not been fully identified and understood.

In preparation for adopting ERAS as a holistic approach, we aimed to assess fast-track (FT) extubation after MICS at our center. In detail, we investigated the potential benefits of on-table extubation outside of a standardized ERAS program. To identify benefits, pitfalls, and gaps, we further focused on a low-risk patient cohort with short postoperative ventilation times.

## Material and methods

The retrospective study included patients who underwent MICS for valvular or cardiac tumor resection via right anterolateral thoracotomy between August 2019 and August 2023, and who were extubated within six hours postoperatively. The patients did not undergo a preoperative educational process as described in an established ERAS program (9). Anesthesia was induced with sufentanil, propofol, and rocuronium, and maintained with remifentanil and sevoflurane. Criteria for extubation were as previously described (10), without the use of an extubation prediction score as described by Subramaniam et al. (11). In brief, patients had to fulfill general practice standards for extubation and further show low medical hemodynamic support as well as low chest tube drainage. Additionally, an adequate response to neurological and respiratory tests was mandatory prior to extubation (10, 12). In addition to opioid titration, we began routinely applying loco-regional anesthesia with ropivacaine in August 2022. The surgeon administered the anesthetic circumferentially around the access site—at the costal arch, muscular, and subcutaneous levels—before thorax closure. Surgical thoracic access site was parasternal, trans-axillary, or anterolateral right, and extracorporeal circulation cannulas were inserted femorally in cut-down technique or percutaneously using a closure device (Perclose™ ProGlide™/Perclose™ ProStyle™, Abbott Laboratories). Emergency surgeries were excluded. Postoperatively, all patients were transferred to ICU. The first visit of physiotherapy was on postoperative day (POD) 1. Potential transfer to the general ward (GW) was possible thereafter. All patients received i.v. unfractionated heparin (UFH) until International Normalized Ratio (INR) using phenprocoumon was >2. Anticoagulation with phenprocoumon for three months postoperatively was started once the pacing wires were removed. If patients were transferred to a peripheral referring hospital postoperatively, the surgical treatment had to be completed, including removal of chest tubes and pacing wires.

In the presented retrospective analysis FT patients who were extubated within six hours in ICU (fast-track group, FT) were compared to those extubated in the OR (extubation in tabula group, EIT). The primary endpoint was FTF, a composite of possible reversals in the postoperative course: revision surgery, re-intubation, and readmission to ICU or IMC. Secondary endpoints encompassed several key outcomes: Major adverse cardiovascular and cerebrovascular events (MACCE) were defined as a composite of cardiovascular death, postoperative myocardial infarction, stroke, and revision surgery. Postoperative pneumonia was identified based on pulmonary infiltrates visible on x-ray, accompanied by elevated systemic infection markers and the requirement for antibiotic treatment. New onset of postoperative atrial fibrillation (POAF) was further included and defined by the need for either medical or electric cardioversion. Additionally, postoperative delirium was evaluated using the Confusion Assessment Method for the Intensive Care Unit (CAM-ICU). Process optimization aspects as well as length of stays were also analyzed and documented.

Statistical analysis was performed using SPSS (Version 29.0.1.1; IBM Corporation). Propensity score matching was achieved by 1:1 best neighbor matching without double matching, based on preoperative baseline characteristics as covariates. Standardized differences are presented in the Supplementary Material (Supplementary Table S1). Normal distribution was assessed by Kolmogorov–Smirnov testing thereafter. Continuous variables are reported as median + Interquartile range (IQR). Categorical data are presented as percentages of the cohort. After matching, paired *t*-tests and McNemar's test were conducted. Subsequently, a multivariate analysis of the unmatched cohort was performed to identify predictive risk factors for FTF. *P*-values < 0.05 were deemed statistically significant.

This retrospective analysis was approved by the local ethics committee (EK23-263) and complies with the Declaration of Helsinki.

## Results

A total of 146 patients underwent MICS during the 4-year study period from 2019–2023. Of these, 44.5% were excluded due to ventilation times exceeding six hours. The remaining 55.5% (*n* = 81) were extubated within six hours postoperatively, adhering to a recommended fast-track protocol, and were therefore included in the study (4, 5). Within this sub-cohort, 55.6% were extubated in the operating room (extubation in tabula, EIT) before being admitted to the ICU, while the remaining 44.4% (fast-track, FT) were admitted to the ICU intubated and extubated within a maximum of six hours. Propensity score matching was performed using preoperative baseline characteristics as covariates, employing 1:1 best neighbor matching to create an analysis group of *n* = 60.

**Figure 1:**
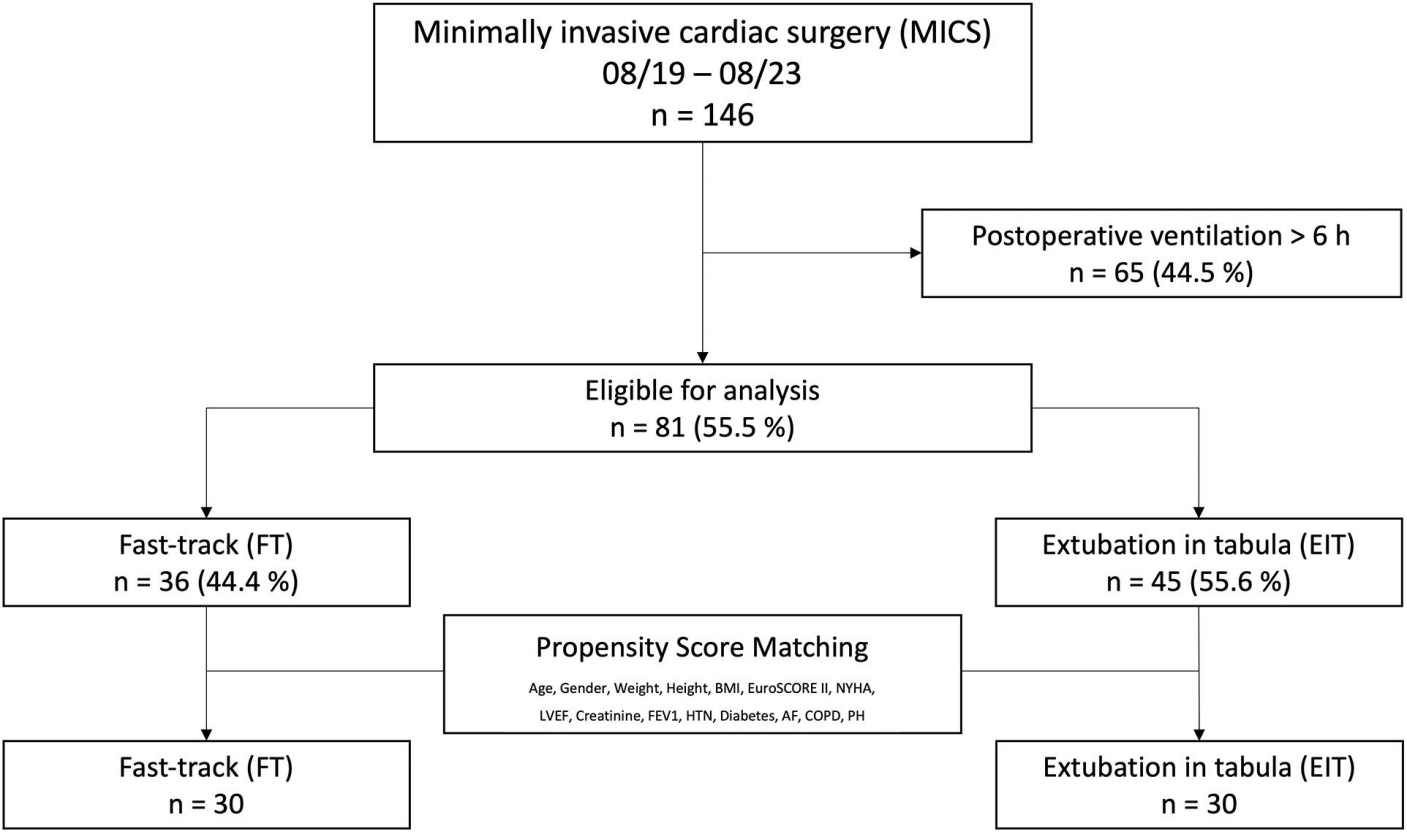
A total of 146 patients underwent minimally invasive cardiac surgery (MICS) via right-sided thoracotomy over a 48-month period. Patients extubated within six hours postoperatively (fast-track, FT) were compared to those extubated in the OR (extubation in tabula, EIT). Patients with ventilation times exceeding six hours were excluded.

Patients who were admitted to ICU intubated, but extubated within six hours after surgery (fast-track, FT) were then compared to those who were extubated in the OR (extubation in tabula, EIT) as shown in Figure 1.

### Baseline and surgical characteristics

The patients had a median age of 61 years, were 76.7% male, and had similar baseline characteristics, notably an overall low EuroSCORE II (median: 0.71; IQR: 0.57–0.91) and no significant differences in additional comorbidities (Table 1). Cardiac, renal, and pulmonary function were comparable in both cohorts.

**Table 1:** Baseline characteristics.

	Overall Cohorts			Propensity Score Matched Cohorts		
	FT	EIT	*p*-value	FT	EIT	*p*-value
	*n* = 36	*n* = 45		*n* = 30	*n* = 30	
Age (y)	62.0 (55.3–68.8)	60.0 (50.0–68.0)	0.527	62.0 (55.3–67.5)	60.5 (50.0–68.0)	0.975
Male, % (*n*)	75.0 (27)	68.9 (31)	0.624	76.7 (23)	76.7 (23)	1.000
Weight (kg)	79.5 (72.0–89.5)	77.0 (68.0 86.0)	0.559	81.5 (69.8–91.0)	79.0 (73.5–86.0)	0.823
Height (cm)	177 (165–181)	175 (170–180)	0.794	177 (167–183)	176 (170–179)	0.800
BMI (kg/m^2^)	25.3 (23.2–30.0)	24.5 (22.8–28.0)	0.311	25.3 (23.3–30.1)	24.7 (22.8–28.1)	0.617
EuroSCORE II (%)	0.78 (0.57–0.93)	0.69 (0.60–0.99)	0.651	0.74 (0.56–0.91)	0.70 (0.59–0.95)	0.596
NYHA > 2, % (*n*)	30.6 (11)	11.1 (5)	0.048	23.3 (7)	13.3 (4)	0.375
LVEF (%)	60.0 (55.5–60.0)	60.0 (55.0–60.0)	0.500	60.0 (56.5–60.0)	60.0 (56.8–60.3)	0.835
Creatinine (mg/dl)	0.9 (0.6–1.0)	0.9 (0.8–1.0)	0.756	0.9 (0.9–1.0)	0.9 (0.8–1.0)	0.574
FEV1 (l)	2.8 (2.0–3.4)	3.0 (2.4–3.7)	0.251	2.8 (2.0–3.4)	2.8 (2.4–3.5)	0.648
HTN, % (*n*)	44.4 (16)	44.4 (20)	1.000	46.7 (14)	53.3 (16)	0.791
Diabetes, % (*n*)	8.3 (3)	6.7 (3)	1.000	6.7 (2)	3.3 (1)	1.000
AF, % (*n*)	22.2 (8)	13.3 (6)	0.379	26.7 (8)	16.7 (5)	0.508
COPD, % (*n*)	5.6 (2)	8.9 (4)	0.688	6.7 (2)	10.0 (3)	1.000
PH, % (*n*)	27.8 (10)	31.1 (14)	0.810	30.0 (9)	33.3 (10)	1.000
MVR, % (*n*)	63.9 (23)	71.1 (32)	0.633	63.3 (19)	66.7 (20)	1.000
AVS, % (*n*)	30.6 (11)	13.3 (6)	0.098	30.0 (9)	16.7 (5)	0.388
Cardiac tumor, % (*n*)	2.8 (1)	13.3 (6)	0.125	3.3 (1)	16.7 (5)	0.219

Baseline characteristics of the analyzed cohorts before and after 1:1 propensity score matching. Results are shown as median + Interquartile range (IQR, 25th—75th).

AF, atrial fibrillation; AVS, aortic valve stenosis; BMI, body mass index; COPD, chronic obstructive pulmonary disease; EIT, extubation in tabula; FEV1, forced expiratory volume; FT, fast-track; HTN, arterial hypertension; LVEF, left ventricular ejection fraction; MVR, mitral valve regurgitation; PH, pulmonary hypertension.

Surgical characteristics are shown in Table 2. Mitral valve repair was performed in 56.7% of all patients. Aortic valve replacement was the second most common surgery (25.0%). Concomitant surgeries, i.e., left atrial appendage closure (LAAO), were performed in 15.0% of the cases. An automated suture fastening system (Cor-Knot®, LSI SOLUTIONS®) was used in 11.7% of surgeries, a percutaneous closure device in 20.0% of cases. FT patients had longer cardiopulmonary bypass [CPB; FT 165.0 min (146.5–217.5); EIT 158.5 min (128.0–189.5); *p* = 0.047], cross-clamping [FT 121.0 min (98.0–142.5); EIT 103.0 min (85.3–122.5); *p* = 0.012], and reperfusion times [FT 32.0 min (26.5–43.0); EIT 28.5 min (21.0–35.8); *p* = 0.016], but the total duration of surgery was comparable [FT 241.5 min (221.5–321.5); EIT 247.0 min (211.0–295.0); *p* = 0.210].

**Table 2:** Surgical characteristics.

	FT	EIT	*p*-value
	*n* = 30	*n* = 30	
MV repair, %	53.3 (16)	60.0 (18)	0.815
MV replacement, %	10.0 (3)	6.7 (2)	1.000
TV repair, %	6.7 (2)	3.3 (1)	1.000
AV replacement, % (n)	33.3 (10)	16.7 (5)	0.267
Concomitant surgery			
LAAO, %	6.7 (2)	0.0 (0)	0.500
Ablation, %	0.0 (0)	6.7 (2)	0.500
PFO Closure, %	10.0 (3)	10.0 (3)	1.000
MICS devices			
PCD, %	16.7 (5)	23.3 (7)	0.727
ASFS, %	10.0 (3)	13.3 (4)	1.000
DOS (min)	241.5 (221.5–321.5)	247.0 (211.0–295.0)	0.210
CPB (min)	165.0 (146.5–217.5)	158.5 (128.0–189.5)	**0** **.** **047**
X-Clamping (min)	121.0 (98.0–142.5)	103.0 (85.3–122.5)	**0** **.** **012**
Reperfusion (min)	32.0 (26.5–43.0)	28.5 (21.0–35.8)	**0** **.** **016**
Cardioplegia (Bretschneider), %	86.2 (25)	76.7 (23)	0.508
Amount (ml)	1,600 (1,500–2,000)	1,600 (1,500–1,600)	0.051
Intraoperative hypothermia (°C)	32.0 (32.0–32.0)	32.0 (32.0–34.0)	0.055
Cannula Size			
Arterial	22.0 (20.0–22.0)	20.0 (18.3–22.0)	0.431
Venous	25.0 (25.0–26.0)	25.0 (25.0–27.0)	0.861

Surgical characteristics of FT group and EIT group. Results are shown as median + Interquartile range (IQR, 25th—75th).

FT, fast-track; EIT, extubation in tabula; MV, mitral valve; TV, tricuspid valve; AV, aortic valve; LAAO, left atrial appendage occlusion; PFO, patent foramen ovale; PCD, percutaneous closure device; ASFS, automated suture fastening system; DOS, duration of surgery; CPB, cardiopulmonary bypass.

*p*-values below 0.05 are shown in bold.

### Postoperative course

All patients were transferred to ICU postoperatively. The median time of initial ventilation in the FT group was 5 h (IQR: 3–6 h). Overall, 29.8% of patients required noninvasive ventilation (NIV) therapy after extubation, with no significant difference between the two cohorts (EIT: 33.3%, FT: 25.9%; *p* = 0.576). At arrival on ICU, core body temperature of FT and EIT patients significantly differ [FT 36.4°C (35.8–37.1); EIT 36.8°C (36.5–37.1); *p* = 0.024]. Patients in both groups reported mild pain based on the numeric rating scale (NRS) during the first assessment on the day of surgery [FT 2 (0–2); EIT 0 (0–2); *p* = 0.179]. Seven scores were recorded within the first hour after arrival, with six of these patients reporting no pain (NRS 0). The application of loco-regional anesthesia with ropivacaine, which was initiated in August 2022, did not show a significant impact on postoperative pain perception [before Aug. `22: 2 (0–2); after Aug. `22: 0 (0–2); *p* = 0.808]. The amount of chest tube drainage until 6:00am on POD 1 was comparable in both groups [FT 330 ml (200–455); EIT 280 ml (198–501); *p* = 0.493]. The median time until the removal of the last chest tube was 3d (IQR: 2–4) postoperatively. EIT patients required significantly less inotropic and vasoactive medication, measured as the postoperative peak of Vasoactive Inotropic Score [VIS; FT 0.50 (0.00–4.93); EIT 7.50 (4.40–12.15); *p* < 0.001] (13, 14). Postoperative lactate levels were significantly higher in patients who remained intubated postoperatively [FT 2.50 mmol/L (1.30–3.88); EIT 1.55 mmol/L (1.05–2.30); *p* = 0.005].

### Primary composite endpoint and secondary outcomes

The primary study endpoint was FTF (Table 3), a composite of revision surgery, re-intubation, and readmission to ICU or IMC. This endpoint occurred in 20.0% of all patients, with a 50% lower incidence in the EIT cohort compared to the FT cohort (FT: 26.7%, EIT: 13.3%; *p* = 0.289). EIT patients underwent half as many revision surgeries (FT 20.0%; EIT 10.0%; *p* = 0.219), though the difference was not statistically significant. Major factors for FTF and revision procedures were the need for postoperative pacemaker implantation (*n* = 3) and haemothorax with major bleeding (*n* = 5).

**Table 3:** Primary composite endpoint and secondary outcomes.

	FT	EIT	*p*-value
	*n* = 30	*n* = 30	
A: Primary Composite Endpoint			
Fast-track failure, % (*n*)	26.7 (8)	13.3 (4)	0.289
Revision surgery, % (*n*)	20.0 (6)	10.0 (3)	0.219
Pacemaker implantation, % (*n*)	10.0 (3)	0.0 (0)	0.250
Haemothorax, % (*n*)	6.7 (2)	10.0 (3)	1.000
Re-intubation, % (*n*)	16.7 (5)	10.0 (3)	0.625
Readmission to ICU, % (*n*)	6.7 (2)	6.7 (2)	1.000
Readmission to IMC, % (*n*)	6.7 (2)	3.3 (1)	1.000
B: Secondary Outcomes			
MACCE, % (*n*)	16.7 (5)	10.0 (3)	0.625
Delirium, % (*n*)	10.0 (3)	6.7 (2)	1.000
Pneumonia, % (*n*)	3.3 (1)	6.7 (2)	1.000
POAF, % (*n*)	26.7 (8)	23.3 (7)	1.000
Length of stay			
ICU (h)	21.5 (20.8–25.5)	22.0 (19.8–24.8.)	0.948
IMC (h)	24.5 (0.0–51.0)	23.5 (0.0–37.6)	0.382
GW (d)	7.0 (5.0–11.0)	7.0 (5.0–9.0)	0.809

**A:** Types of fast-track failure (FTF) in FT and EIT patients **B:** Analysis of secondary outcomes. Results are shown as percentages of each cohort. Continuous variables are shown as median + Interquartile range (IQR, 25th—75th).

FT, fast-track; EIT, extubation in tabula; GW, general ward; ICU, intensive care unit; IMC, intermediate care unit; MACCE, major adverse cardiovascular cerebrovascular events; POAF, postoperative atrial fibrillation.

When comparing patients with FTF to patients, who underwent a linear postoperative course (Control, CTRL), baseline characteristics were comparable. The type of surgery, surgical characteristics (duration of surgery, CPB time, cross-clamping time, time of reperfusion), and concomitant procedures did not differ between FTF and CTRL patients (Supplementary Table S2). However, intraoperative core body temperature was significantly lower in FTF patients [CTRL 32.0°C (32.0–34.0); FTF 32.0°C (29.0–32.0); *p* = 0.022]. In the early postoperative course, VIS levels [CTRL 3.95 (0.00–6.80); FTF 8.30 (4.60–16.43); *p* = 0.033] differed significantly, while ventilation times [CTRL 0 h (0–5); FTF 4 h (0–6); *p* = 0.151] and lactate levels [CTRL 1.65 mmol/L (1.13–2.50); FTF 3.00 mmol/L (1.23–5.00); *p* = 0.067] were only numerically different. As shown in Table 4, when conducting a multivariate analysis (logistic regression) of the unmatched cohort with baseline characteristics as covariates, atrial fibrillation could be identified as a factor that predicts postoperative FTF [OR: 12.77 (1.48, 109.95), *p* = 0.020]. However, the wide 95% confidence interval should not be overlooked. When objective intraoperative characteristics were additionally considered in a second analysis, intraoperative hypothermia emerged as the primary contributor to FTF [OR: 0.24 (0.07, 0.89), *p* = 0.033].

**Table 4:** Baseline characteristics as potential predictors for fast-track failure.

	OR	95% CI	*p*-value
Age (y)	1.02	(0.92, 1.13)	0.710
Male, % (*n*)	1.06	(0.09, 12.08)	0.965
Weight (kg)	1.02	(0.46, 2.24)	0.968
Height (cm)	0.92	(0.46, 1.82)	0.808
BMI (kg/m^2^)	0.98	(0.10, 9.65)	0.983
EuroSCORE II (%)	0.01	(0.00, 1.41)	0.067
NYHA > 2, % (*n*)	1.06	(0.12, 9.12)	0.957
LVEF (%)	0.97	(0.84, 1.11)	0.626
Creatinine (mg/dl)	3.90	(0.03, 461.47)	0.576
FEV1 (l)	0.93	(0.21, 4.14)	0.924
HTN, % (*n*)	1.06	(0.17, 6.63)	0.950
Diabetes, % (*n*)	3.67	(0.18, 75.84)	0.400
AF, % (*n*)	12.77	(1.48, 109.95)	**0** **.** **020**
COPD, % (*n*)	2.59	(0.14, 47.90)	0.522
PH, % (*n*)	4.71	(0.53, 42.16)	0.166
DOS (min)	1.00	(0.95, 1.06)	0.916
CPB (min)	1.08	(0.95, 1.22)	0.230
X-Clamping (min)	0.94	(0.83, 1.06)	0.334
Reperfusion (min)	0.86	(0.72, 1.03)	0.104
Intraoperative hypothermia (°C)	0.24	(0.07, 0.89)	**0** **.** **033**

Multivariable analysis including all variables used for the propensity score matching process. Results are presented about the association of FTF to preoperative baseline characteristics (top). Surgical parameters were included in a second analysis along with baseline characteristics (bottom).

AF, atrial fibrillation; BMI, body mass index; CI, confidence interval; COPD, chronic obstructive pulmonary disease; CPB, cardiopulmonary bypass; DOS, duration of surgery; EIT, extubation in tabula; FEV1, forced expiratory volume; FT, fast-track; HTN, arterial hypertension, LVEF, left ventricular ejection fraction; PH, pulmonary hypertension.

*p*-values below 0.05 are shown in bold.

Further postoperative complications (MACCE, delirium, pneumonia, POAF) occurred in 51.7% of all patients, who were extubated within six hours postoperatively, regardless of the definition of FTF. There was no significant difference when comparing FT and EIT.

Furthermore, postoperative milestones were analyzed. Time from skin closure to first ICU blood gas analysis was similar between FT and EIT patients [FT 49.5 min (44.0–62.3); EIT 52.0 min (44.8–62.3); *p* = 0.835]. Median ICU, IMC, and GW stays were 22 h, 24 h, and 7 days, respectively. Discharge destination, INR at discharge and days since removal of the last chest tube were comparable in both groups.

## Discussion

This retrospective single-center analysis demonstrates the feasibility and safety of extubation in the operating room following MICS. The primary endpoint, fast-track failure (FTF), occurred in 20.0% of patients (FT: 26.7%, EIT: 13.3%; *p* = 0.289), showing non inferiority of EIT after MICS. FT patients exhibited longer CPB, cross-clamping, and reperfusion times, higher postoperative lactate levels, and greater hemodynamic support. Secondary outcomes, including MACCE, delirium, pneumonia, and POAF, were comparable between the cohorts. EIT did not significantly affect ICU, IMC, or total hospital length of stay. These results suggest that the primary benefit of extubation in the operating room after MICS arises during the postoperative period in the ICU, where intensified patient-oriented physiotherapy—as recommended by the ERAS Cardiac Society (4)—can be implemented. Since MICS promotes thoracic stability, facilitating early mobilization and shorter hospital stays, and ERAS pursues similar goals by encouraging early extubation, mobilization, and transfer to the general ward (3–5), the combination of both approaches may be highly beneficial. While trials have examined extubation strategies, data on reduced ventilation time through EIT in MICS remain limited (7, 8).

### Patient selection

The ERAS Cardiac Society recommends a postoperative ventilation time of less than six hours (4, 6). However, it is challenging to determine whether prolonged ventilation is due to preoperative and intraoperative factors or hospital-specific practices. Additionally, it is difficult to prove if any benefits are related to extubation management. While Probst et al. demonstrated early extubation benefits in coronary artery bypass grafting (CABG) (15), evidence for MICS is limited (7, 8). We hypothesize that patients extubated within six hours could also be safely extubated in the OR if this is established as a goal at the beginning of the surgery, leading us to analyze patients according to ERAS Cardiac Society guidelines (4, 5).

### Peri- and intraoperative management

Identifying FTF risk factors is crucial for safe immediate extubation and early transfer. FT and EIT as well as CTRL and FTF patients were statistically comparable regarding preoperative baseline characteristics. Yet, atrial fibrillation seems to be more present in FTF patients. Although the small sample size must be considered, our logistic regression model also indicates that preoperative atrial fibrillation significantly increases the odds of FTF [OR: 12.77 (1.48, 109.95), *p* = 0.020]. Indeed, two patients in the shown cohorts needed to be readmitted to ICU due to arrythmia. Prophylactic magnesium (16) and standardized intravenous amiodarone could be included in ERAS protocols for mitral valve surgery, as shown by Kar et al. (17). Recently updated guidelines recommended intraoperative surgical ablation without additional mortality risk (Class I, Level A) (18), thus surgical ablation should also be considered in each case.

Core body temperature may influence ventilation times as well as FTF risk. In the presented cohorts FT patients showed significantly lower postoperative core body temperatures [FT 36.4°C (35.8–37.1); EIT 36.8°C (36.5–37.1); *p* = 0.024]. Additionally, according to the subsequently conducted multivariate analysis based on unmatched preoperative characteristics, surgical times and intraoperative core body temperature, intraoperative hypothermia emerged as a potential contributor to FTF [OR: 0.24 (0.07, 0.89), *p* = 0.033]. Thus, effective temperature management is essential for ICU care and immediate extubation. Two major factors in temperature management are the cardioplegic solution and early rewarming. Crystalloid-based cardioplegia is often administered cold in a single dose, while whole-blood cardioplegia can also be given at 34°C in intervals (19, 20). Both solutions offer comparable myocardial protection (20). However, avoiding hyperthermia during cardiopulmonary bypass rewarming is critical to prevent neurologic injury and renal failure in both cases (21, 22). Therefore, we suggest modified whole-blood cardioplegia with early rewarming (e.g., at the time when beginning valve/ring knotting) in the setting of MICS and ERAS at the same time. Interdisciplinary communication between perfusionists, anesthetists, and surgeons is essential.

Van Praet et al. identified prolonged CPB time as a predictor of postoperative FTF (23), consistent with Malvindi et al., who linked longer CPB times to non-fast-track extubation (8). In our FT cohort, both cardiopulmonary bypass time and reperfusion time were significantly longer. Although the DOS was also longer, the difference did not reach statistical significance, which may be attributable to the smaller sample size. However, Malvindi et al.'s reported benefit of early extubation was not observed when comparing OR extubation to extubation within six hours postoperatively, which is in line with our findings (8). Although previous studies (8), including our own cohorts, have reported significantly shorter aortic cross-clamping times in EIT patients, our logistic regression model indicates that aortic cross-clamping duration does not significantly contribute to FTF. However, intraoperative hypothermia appears to play a role, as demonstrated above. Additionally, the duration of reperfusion showed an odds ratio of 0.86 with a confidence interval of 0.72–1.03 (*p* = 0.104), suggesting a potential trend considering the small sample size of our cohorts.

### Safety and efficacy: fast-track failure

The primary endpoint of fast-track failure (FTF) was met in 12 patients, with no significant difference between the FT and EIT groups, although it occurred numerically twice as often in the FT cohort (FT: 26.7%, EIT: 13.3%; *p* = 0.289). Revision surgery accounted for 73.8% of FTF, more frequent in the FT cohort. While our FTF findings align with existing literature, EIT had little impact on the postoperative course (8, 23, 24). Extubation in the OR did not affect ICU, IMC, or total length of hospital stay, with both groups showing similar IMC avoidance. Malvindi et al. noted longer ICU stays for those intubated over six hours, though this difference was also partially vanished in subgroups of EIT and FT (8). Our cohort's non-superiority may be due to hospital-specific perioperative management, where patients remained in ICU without early mobilization. EIT could shorten stays if early transfer including same-day physiotherapy is prioritized as shown by Moradian et al. (25) in a cohort of patients undergoing CABG.

Furthermore, therapeutic anticoagulation also limited early discharge, with no significant differences in INR levels or discharge destinations in our cohort. Chest tubes were typically removed on POD 3, with UFH bridging starting thereafter. Considering subcutaneous LMWH could facilitate shorter stays. Current guidelines suggest UFH and LMWH are equally viable for patients with biological mitral valves (26). For mechanical valves, LMWH is off-label but promising results have already been shown suggesting similar efficacy (26).

### Implementation of a standardized ERAS program

**Figure 2:**
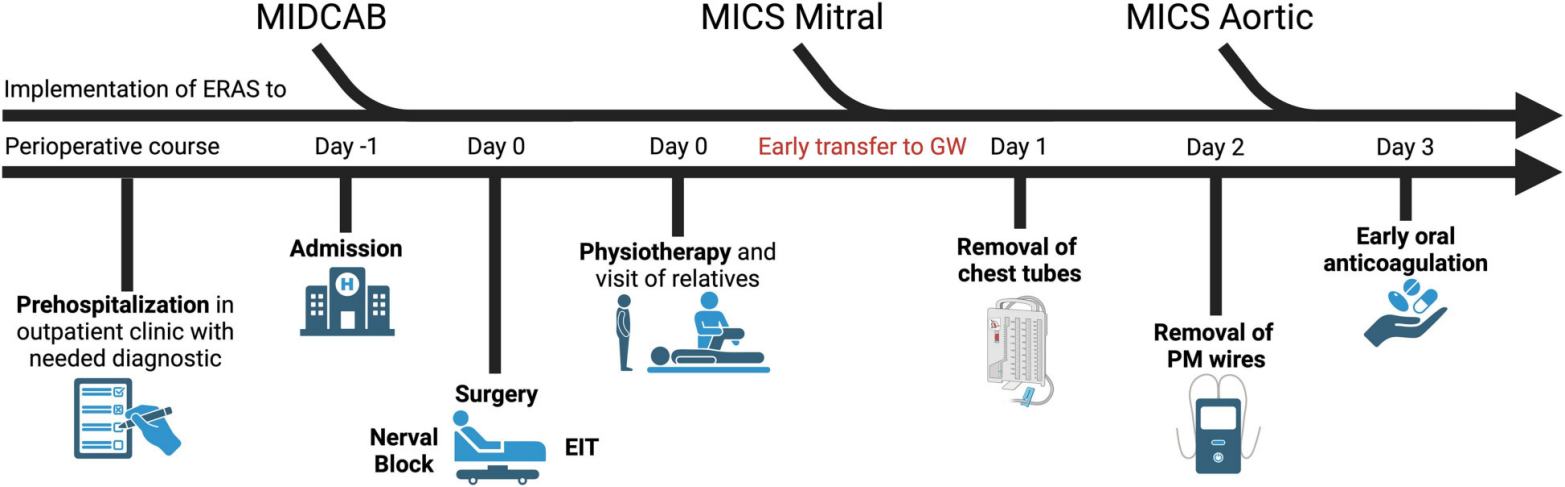
Suggested protocol for implementation of an enhanced recovery after surgery (ERAS) program at a new institution. MIDCAB, minimally invasive direct coronary artery bypass; MICS, minimally invasive cardiac surgery; EIT, extubation in tabula; GW, general ward; PM, pacemaker. The figure was created with biorender.com.

The retrospective analysis highlights the potential benefits of EIT for low-risk patients undergoing MICS. EIT is safe, requiring less inotropic and vasoactive medication, with no significant differences in ICU, IMC, and GW stays. We believe that significant improvements can be achieved when early mobilization and return to daily activities are prioritized, as in an established ERAS program. We propose Figure 2 as a guide for implementing an ERAS program, starting with MICS in coronary surgery (minimally invasive direct coronary artery bypass, MIDCAB) due to shorter operative times and less need for cooling and CPB, before progressing to mitral and aortic cases. Notably, when ERAS is used for MIDCAB, there is no need to switch to a single-lumen tube if a left-sided double-lumen tube is used during surgery (27). Patients should undergo complete preoperative diagnostics and prehospitalization a day prior to surgery. Local analgesics should be administered at the end of surgery. Further components should include extubation in the OR, same-day physiotherapy, and family visits to minimize postoperative delirium risk (4). Oral anticoagulation should be started at the earliest possible point.

### Limitations of the study

This study is limited by its small sample size, retrospective nature, and single-center design, making findings applicable only to low-risk patients undergoing MICS. Patients were retrospectively selected if ventilation time did not exceed 6 h, which also underscores that, while ERAS is established in many centers, there remains no universal pre- and intraoperative standard for patient selection for ERAS as well as EIT. Results of the conducted multivariate analysis should therefore be interpreted keeping in mind the sample size of *n* = 81 patients in the unmatched cohort. There was no follow-up time, so long-term effects cannot be assessed. Nonetheless, the study demonstrated potential benefits of EIT on ICU stay and overall hospital length of stay. These findings will be further explored within an established ERAS protocol.

## Conclusion

Based on our retrospective single-center experience with a matched cohort of 60 patients, extubation in the operating room during minimally invasive cardiac surgery appears feasible and safe, even outside a structured ERAS program. Adequate intraoperative temperature management seems to be a major contributor to both extubation in the OR and postoperative FTF. FTF occurred numerically more often in intubated patients, often due to revision surgeries like pacemaker implantation or hemothorax, though not statically significant. While EIT did not significantly impact time in various units (ICU, IMC, GW), transfers to step-down units occurred earlier. Despite its mentioned limitations, this study highlights the necessity of a multimodal approach to enable patients to benefit from early extubation (e.g., same-day physiotherapy). Additionally, this further demonstrates the need for standardized preoperative criteria for patient selection, not only for ERAS but also for EIT. These results are expected to improve within a prospective ERAS program.

## Data Availability

The raw data supporting the conclusions of this article will be made available by the authors, without undue reservation.
