# Supplementary material for: Fast-track extubation in minimally invasive cardiac surgery: limits and lessons of a 4-year single-center analysis
Source: Front Cardiovasc Med. 2025 Sep 8;12:1567533. doi: 10.3389/fcvm.2025.1567533 (PMC12450875; doi:10.3389/fcvm.2025.1567533)
Supplement: Supplementary file 1 [file Table1.docx]

| **Supp. 1: Standardized Differences** | | |
| --- | --- | --- |
|  | **Before PSM** | **After PSM** |
| Age | 8,9 | 0,8 |
| Male | 13,6 | 0,0 |
| Weight | 16,9 | 6,8 |
| Height | 7,8 | 6,4 |
| BMI | 26,0 | 13,6 |
| EuroSCORE II | 14,9 | 15,6 |
| NYHA > 2 | 49,4 | 26,1 |
| LVEF | 8,2 | 6,3 |
| Creatinine | 0,0 | 14,5 |
| FEV1 | 28,4 | 11,5 |
| HTN | 0,0 | 13,2 |
| Diabetes | 6,1 | 15,6 |
| AF | 23,5 | 24,4 |
| COPD | 12,8 | 12,0 |
| PH | 7,2 | 7,1 |
| Standardized differences (%) of the analyzed cohorts before and after 1:1 propensity score matching. Abbreviations: AF – atrial fibrillation, BMI – body mass index, COPD – chronic obstructive pulmonary disease, FEV1 – Forced expiratory volume, HTN – arterial hypertension, LVEF – left ventricular ejection fraction, PH – pulmonary hypertension, PSM – Propensity Score Matching. | | |

| **Supp. 2: Analysis of baseline characteristics and perioperative parameters of fast-track failure patients** | | | |  |
| --- | --- | --- | --- | --- |
|  | **CTRL**  **n=48** | **FTF**  **n=12** | **p-value** |  |
| Extubation in tabula | 54.2 (26) | 33.3 (4) | 0.333 |  |
| **A: Baseline characteristics** |  |  |  |  |
| Age (y) | 60.0 (50.0 – 66.8) | 66 (53.3 – 70.5) | 0.240 |  |
| Male, % (n) | 77.1 (37) | 75.0 (9) | 1.000 |  |
| Weight (kg) | 82.0 (70.5 – 87.0) | 76.5 (72.8 – 85.0) | 0.664 |  |
| Height (cm) | 177 (177 – 182) | 176 (160 – 176) | 0.160 |  |
| BMI (kg/m^2^) | 25.1 (22.5 – 29.5) | 25.2 (24.4 – 28.9) | 0.548 |  |
| EuroSCORE II (%) | 0.70 (0.56 – 1.04) | 0.74 (0.62 – 0.87) | 1.000 |  |
| NYHA > 2, % (n) | 18.8 (9) | 16.7 (2) | 1.000 |  |
| LVEF (%) | 60.0 (57.0 – 60.0) | 60.0 (55.8 – 60.0) | 0.770 |  |
| Creatinine (mg/dl) | 0.9 (0.8 – 1.0) | 0.9 (0.8 – 1.0) | 0.850 |  |
| FEV1 (l) | 2.81 (2.30 – 3.58) | 2.80 (1.83 – 3.20) | 0.256 |  |
| HTN, % (n) | 47.9 (23) | 58.3 (7) | 0.748 |  |
| Diabetes, % (n) | 4.2 (2) | 8.3 (1) | 0.495 |  |
| AF, % (n) | 16.7 (8) | 41.7 (5) | 0.110 |  |
| COPD, % (n) | 8.3 (4) | 8.3 (1) | 1.000 |  |
| PH, % (n) | 29.2 (14) | 41.7 (5) | 0.493 |  |
| **B: Perioperative parameters** | |  |  |  |
| MV repair, % (n)  Ring diameter (mm)  Neochordae (n) | | 60.4 (29)  34.0 (32.5 – 36.0)  2.0 (1.0 – 3.0) | 41.7 (5)  34.0 (32.0 – 37.0)  1.9±1.2 | 0.332  0.979  0.747 |
| MV replacement, % (n)  Prosthesis diameter (mm) | | 4.2 (2)  31.0 (23.3 – 31.3) | 25.0 (3)  32.0 (31.0 – 33.0) | **0.050**  0.197 |
| TV repair, % (n) | | 2.1 (1) | 16.7 (2) | 0.099 |
| AV replacement, % (n)  Prosthesis diameter (mm) | | 25.0 (12)  23.0 (23.0 – 25.0) | 25.0 (3)  23.0 (23.0 – 25.0) | 1.000  0.812 |
| Concomitant surgery  LAAO, % (n)  Ablation, % (n)  PFO Closure, % (n) | | 4.2 (2)  2.1 (1)  8.3 (4) | 0.0 (0)  8.3 (1)  16.7 (2) | 1.000  0.363  0.590 |
| MICS devices  PCD, % (n)  ASFS, % (n) | | 25.0 (12)  14.6 (7) | 0.0 (0)  0.0 (0) | 0.101  0.326 |
| DOS (min) | | 243.0 (211.3 – 303.5) | 253.5 (230.3 – 301.0) | 0.380 |
| CPB (min) | | 157.0 (135.0 – 203.5) | 177.5 (153.3 – 216.5) | 0.156 |
| X-Clamping (min) | | 103.0 (91.0 – 126.0) | 122.0 (100.5 – 134.5) | 0.180 |
| Reperfusion (min) | | 29.0 (21.0 – 38.0) | 35.0 (28.5 – 47.3) | 0.164 |
| Cardioplegia (Bretschneider), %  Amount (ml) | | 76.6 (36)  1600 (1500 – 1600) | 100 (12)  1600 (1500 – 2000) | 0.098  0.414 |
| Cannula Size  Arterial (Fr)  Venous (Fr) | | 20.0 (18.5 – 22.0)  25.0 (25.0 – 27.0) | 22.0 (20.0 – 22.0)  25.0 (25.0 – 26.5) | 0.194  0.592 |
| Intraoperative hypothermia (°C) | | 32.0 (32.0 – 34.0) | 32.0 (29.0 – 32.0) | **0.022** |
| Analysis of baseline characteristics and perioperative parameters of fast-track failure (FTF) patients. Results are shown as percentages of each cohort. Continuous variables are shown as median + Interquartile range (IQR, 25^th^ – 75^th^). Abbreviations: AF – atrial fibrillation, ASFS – automated suture fastening system, AV – aortic valve, AVS – aortic valve stenosis, BMI – body mass index, COPD – chronic obstructive pulmonary disease, CPB – cardiopulmonary bypass, CTRL – control, DOS – duration of surgery, EIT – extubation in tabula, FEV1 – Forced expiratory volume, FT – fast-track, HTN – arterial hypertension, LAAO – left atrial appendage occlusion, LVEF – left ventricular ejection fraction, MV – mitral valve, MVR - mitral valve regurgitation, PCD - percutaneous closure device, PFO – patent foramen ovale, PH – pulmonary hypertension, TV – tricuspid valve. | | | | |
